# Exosome secretome and mediated signaling in breast cancer patients with nontuberculous mycobacterial disease

**DOI:** 10.18632/oncotarget.14964

**Published:** 2017-02-01

**Authors:** Julie V. Philley, Anbarasu Kannan, David E. Griffith, Megan S. Devine, Jeana L. Benwill, Richard J. Wallace, Barbara A. Brown-Elliott, Foram Thakkar, Varsha Taskar, James G. Fox, Ammar Alqaid, Hernaina Bains, Sudeep Gupta, Santanu Dasgupta

**Affiliations:** ^1^ Departments of Medicine, The University of Texas Health Science Center at Tyler, Texas, USA; ^2^ Cellular and Molecular Biology, The University of Texas Health Science Center at Tyler, Texas, USA; ^3^ The Mycobacteria/Nocardia Research Laboratory Department of Microbiology, The University of Texas Health Science Center at Tyler, Texas, USA; ^4^ Medical Oncology, Tata Memorial Center, Mumbai, India

**Keywords:** Exosomes, breast cancer, nontuberculous Mycobacterium, ECM1, biomarker

## Abstract

Bronchiectasis Nontuberculous mycobacterium (NTM_nb_) infection is an emerging health problem in breast cancer (BCa) patients. We measured sera exosome proteome in BCa-NTM_nb_ subjects and controls by Mass Spectroscopy. Extracellular matrix protein 1 (ECM1) was detected exclusively in the circulating exosomes of 82% of the BCa-NTM_nb_ cases. Co-culture of ECM1^+^ exosomes with normal human mammary epithelial cells induced epithelial to mesenchymal transition accompanied by increased Vimentin/CDH1 expression ratio and Glutamate production. Co-culture of the ECM1^+^ exosomes with normal human T cells modulated their cytokine production. The ECM1^+^ exosomes were markedly higher in sera obtained from BCa-NTM_nb_ subjects. Exclusive expression of APN, APOC4 and AZGP1 was evident in the circulating exosomes of these BCa-NTM_nb_ cases, which predicts disease prevalence independent of the body max index in concert with ECM1. Monitoring ECM1, APN, APOC4 and AZGP1 in the circulating exosomes could be beneficial for risk assessment, monitoring and surveillance of BCa-NTM_nb_.

## INTRODUCTION

Nontuberculous mycobacterial lung disease (NTM) is a growing health problem in North America and worldwide [1]. NTM lung disease occurs inindividuals with anatomic lung abnormalities, certain genetic disorders, such as cystic fibrosis, and individuals with no known immunologic abnormalities [2]. The frequency of NTM patients with disease characterized radiographically by nodules and bronchiectasis or nodular bronchiectatic disease (NTM_nb_) in particular, has increased over the past few decades [1]. The majority of these cases involved *Mycobacterium avium* complex (MAC) infection and occurs primarily in Caucasian women with a specific body habitus [3–4]. Notably, there is an increasing rate of NTM_nb_ disease in Caucasian women with a current diagnosis or history of breast cancer. However, there are no molecular factors known to be associated with the development of NTM_nb_ disease in these BCa patients.

Exosomes are emerging as the critical determinants of the immune system function, tumorigenesis and host-pathogen interaction during microbial infection [5–10]. Exosomes are small (50-200 nm), secreted and biologically active endocytic vesicles present in all cell types, circulation and body fluids [5–10]. Being enriched in selective nucleic acids and proteins, the exosomes can influence cellular growth, immune function, facilitate infection and are thus promising for biomarker and therapeutic development [5–10].

Through mass spectroscopy analysis, we identified an exclusive expression signature of ECM1 in the majority of the BCa-NTM_nb_ subjects and numerous BCa subjects examined. The ECM1 containing exosomes were markedly higher in BCa-NTM_nb_ subjects compared to the controls. Co-culture of the ECM1 enriched exosomes with non-tumorigenic breast epithelial cells induced EMT changes accompanied by an increased expression ratio of Vimentin/E-Cadherin and glutamate production. Notably, Mycobacterium avium complex (MAC) culture derived exosomes obtained from the sputum of the BCa-NTM_nb_ patients were found to harbor human ECM1 protein. On the other hand, co-culture of the ECM1 containing exosomes with normal human T cells modulated their cytokine production profile within an hour of the culture. Other than ECM1, exclusive expression signature of the lipolysis associated proteins APOC4, APN and AZGP1 was also observed in the majority of the BCa-NTM_nb_ subjects. A molecular signature of ECM1, APOC4, APN and AZGP1 expression in the circulating exosomes predicts disease prevalence independent of the body max index.

## RESULTS

### Exosome proteome analysis

**Figure 1:**
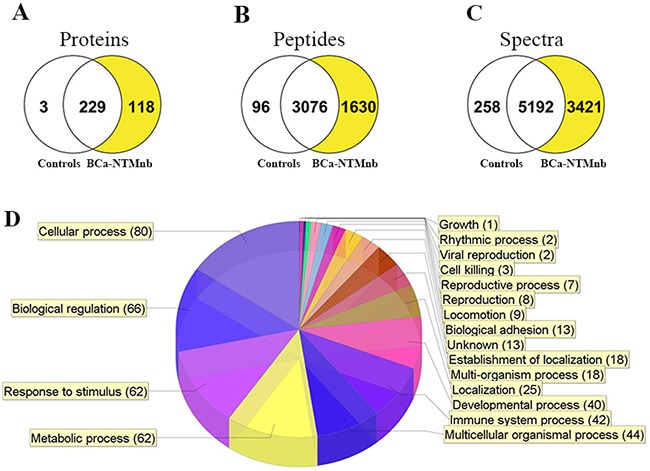
Exosome proteome profiling Venn diagram showing exclusive 118 protein expression **A**. their unique peptides **B**. and spectra **C**. detected in the circulating exosomes of the breast cancer patients with nontuberculous Mycobacterium (BCa-NTM_nb_) infection (yellow shades) compared to the healthy normal and only NTM controls. **D**. Pie chart exhibiting the involvement of the 118 exosome proteins in various biological pathways.

Using Mass spectroscopy, we profiled the exosome content from sera of 17 women with BCa-NTM_nb_, 1 healthy subject and 2 subjects with NTM_nb_. We identified a total of 350 proteins in the exosomes of these above subjects (Figure 1A, Supplementary Table 1). Of these 350 proteins, an exclusive panel of 118 proteins was detected only in the exosomes of the BCa-NTM_nb_ subjects (Figure 1A and Supplementary Table 2). A unique signature of 1630 peptides (Figure 1B) and 3421 spectra (Figure 1C) were evident in the circulating exosomes of the BCa-NTM_nb_ subjects. These proteins are involved in various biological pathways including cell signaling, metabolic and immune system processes (Figure 1D).

### Abundance of ECM1 protein in the circulating exosomes

**Figure 2:**
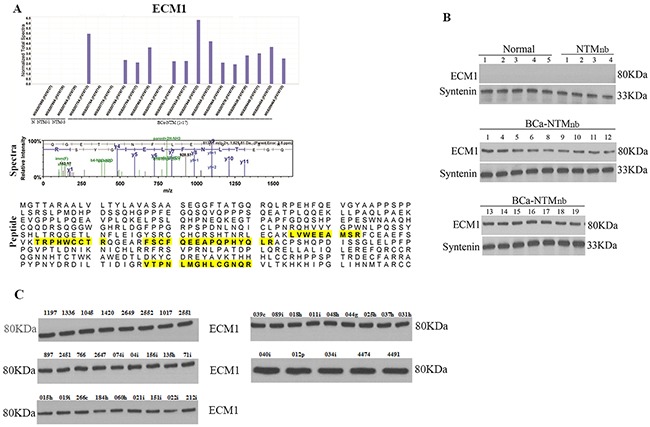
ECM1 detection and validation **A**. Mass spectroscopy analysis detected ECM1 expression only in the breast cancer patients with Bronchiectasis non tuberculous Mycobacterium (BCa-NTM_nb_) infection, but not in the healthy, normal (N1) or individuals with Bronchiectasis non tuberculous Mycobacterium (NTM_nb_) infection. The spectra (panel 2) and 4 unique peptides (shaded yellow, panel 3) of ECM1 detected in the circulating exosomes. **B**. Western blotting analysis in the circulating exosomes of the breast cancer patients with Bronchiectasis non tuberculous Mycobacterium (BCa-NTM_nb_) infection, healthy, normal (N) or individuals with Bronchiectasis non tuberculous Mycobacterium (NTM_nb_) infection. Syntenin was used as an exosome marker and loading control. **C**. Western blot analysis of ECM1 in additional 40 BCa subjects.

Among the 118 unique proteins, extracellular matrix protein 1 (ECM1) expression was detected in 82% (14/17) of the BCa-NTM_nb_ subjects (Figure 2A, Table 1). We did not detect ECM1 expression in the circulating exosomes of the healthy control or subjects with NTM_nb_ (Figure 2A). A number of unique spectra and 4 peptides (Figure 2A) of ECM1 were detected in the circulating exosomes of the 14 BCa-NTM_nb_ subjects. Western blotting was performed to assess ECM1 expression in the sera exosomes of 11 healthy controls, 5 NTM_nb_ and all the 14 positive and an additional 2 BCa-NTM_nb_ subjects (Figure 2B and Supplementary Figure 1A). Exclusive expression of ECM1 was confirmed in 100% (16/16) of the BCa-NTM_nb_ subjects examined (Figure 2B). In addition to Syntenin, we also confirmed the purity of the exosomes in various groups by typing them with CD63 (Supplementary Figure 1B), a commonly used exosome marker [5–10]. We have also compared the exosomal protein expression pattern between the BCa-NTM_nb_ subjects with ECM1 expression (N=14) and without ECM1 expression (N=3) (Figure 1). We observed a unique signature of 110 proteins in the ECM1^+^ group compared to the ECM1^-^ group (Supplementary Figure 2, Supplementary Table 3). A signature of 550 peptides and 665 spectra were noted in the ECM1^+^ BCa-NTM_nb_ group (Supplementary Figure 2A. Notably, these 110 proteins were among the list of 118 identified above (Figure 1, Supplementary Table 2). Being located in different cellular compartments, they are involved in various biological processes through distinct molecular functions (Supplementary Figure 2B-2D, Supplementary Table 3). On the other hand, expression of ECM1 was confirmed in sera exosomes of 40 women with BCa at their various stages (Figure 2C, stages 0-IV). Thus, ECM1 expression was present in BCa subjects alone or BCa subjects with NTM_nb_ disease.

**Table 1:** Demographic information of the BCa-NTM_nb_ subjects with corresponding exosome protein expression

BCa-NTM cases	Age at NTM diagnosis	Age at BCa Diagnosis	BCa Stage	^1^Smoking/Drinking	Weight (lb)	Height (inches)	^2^BMI	^3^ECM1	APN	AZGP1	APOC4
1	75	38	UK	UK	120	63	21.3	+	-	+	-
2	60	65	0	N/N	132	65	22.0	-	-	-	+
3	66	45	I	N/N	143	65	23.8	-	-	+	+
4	57	61	I	N/N	180	69	**25.0**	+	+	-	-
5	71	75	0	N/N	120	64	20.6	+	+	+	+
6	73	68	II	N/Y	150	63	**26.6**	+	-	-	+
7	54	52	II	N/N	132	66	21.3	-	-	-	
8	63	58	I	FS/N	120	67	18.8	+	-	+	
9	61	45	0	FS/Y	145	66	23.4	+	+	-	+
10	55	51	III	N/N	98	61	18.5	+	+	+	+
11	68	68	0	FS/N	156	54	**37.6**	+	-	-	+
12	55	61	IB	N/Y	157	67	24.6	+	+	+	+
13	66	56	II	N/N	91	65	*15.1*	+	+	-	+
14	55	48	I	N/Y	137	68	22.8	+	+	-	-
15	82	76	II	N/N	122	64	20.9	+	+	+	-
16	70	81	0	N/N	175	61	**33.1**	+	+	+	-
17	85	53	I	N/Y	91	63	*16.1*	+	+	+	-
18	73	69	0-I	FS/N	107	61	**30.2**	+	-	-	-
19	69	51	0-I	FS/Y	120	56	**26.9**	+	-	-	-

^1^Histroy of tobacco smoking and alcohol drinking. UK: Unknown; FS: Former smoker; Y: Yes; N: No. ^2^BMI: Body mass index was determined following NHLBI criteria: Underweight = <18.5; Normal weight = 18.5–24.9; Overweight = 25–29.9; Obesity = BMI of 30 or greater. ^3^Expression of the ECM1, APN, AZGP1 and APOC4 in the circulating exosomes.

### The circulating exosomes enriched in ECM1 facilitate epithelial to mesenchymal transition

**Figure 3:**
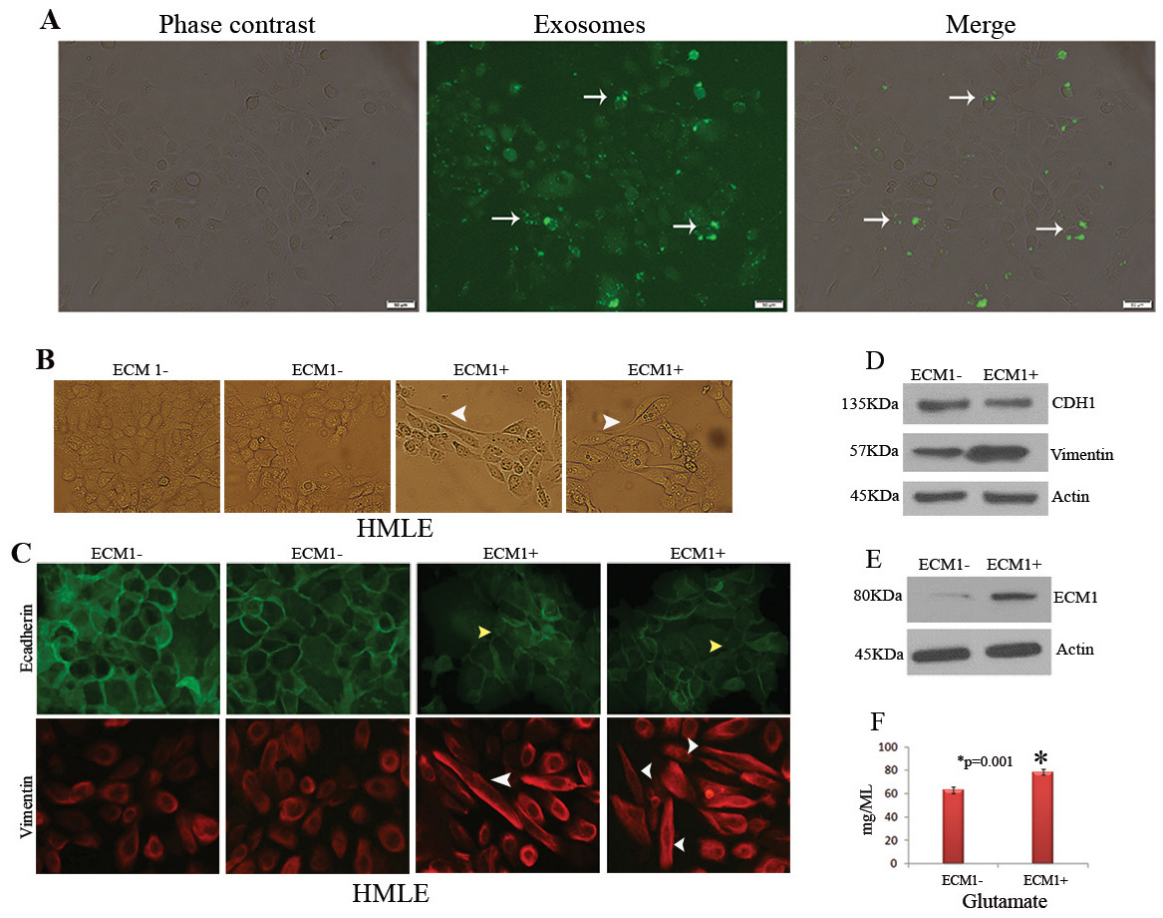
Role of ECM1+ exosomes on cellular transformation **A**. Delivery of the exosome cargo to normal human mammary epithelial cell line HMLE (arrows). Exosomes were purified from sera, labelled with exo-glow green and co-cultured with the HMLE cells for 24 hours followed by imaging under a fluorescent microscope. Magnification X 200. **B**. Marked epithelial to mesenchymal transition (arrows) in the HMLE cells following co-culture with the ECM1^+^ exosomes compared to the ECM1^-^ exosomes. Magnification X 200. **C**. Increased Vimentin/CDH1 ratio (red/green signal intensity) treated cells with disrupted cell-cell contact (yellow arrowheads) and mesenchymal differentiation (white arrowheads) in the cells treated with ECM1^+^ exosome. Magnification X 200. **D**. Western blot analysis confirming decreased expression of CDH1 and augmented expression of Vimentin in HMLE cells treated with ECM1^+^ exosomes. **E**. Higher ECM1 expression in HMLE cells treated with ECM1^+^ exosomes compared to the control. Actin was used as loading control (D-E). **F**. Glutamate production was higher (p=0.001) in the HMLE cells treated with ECM1^+^ exosomes compared to the ECM1^-^ exosomes.

We co-cultured normal human mammary epithelial HMLE cells with ECM1^+^ and ECM1^-^ exosomes purified from the sera of one BCa-NTM_nb_ subject expressing ECM1 (#BCa-NTM_nb_ 10, Table 1). The localization of the exosomes in the HMLE cells was confirmed following 24 hrs. of the co-culture (Figure 3A). The HMLE cells treated with the ECM1^+^ exosomes for 1 week exhibited profound EMT alteration compared to the cells treated with ECM1^-^ exosomes (Figure 3B, arrowheads). We also observed considerable loss of expression of CDH1 and enhanced expression of Vimentin in the HMLE cells treated with the ECM1^+^ exosomes compared to the control by both IF or Western blot analyses (Figure 3C–3D). Increased expression of ECM1 was also confirmed in the ECM1^+^ exosome treated cells (Figure 3E). Disrupted cell-cell contact (Figure 3C, upper panel, yellow arrowheads) and EMT phenotype (Figure 3C, lower panel, white arrowheads) were further confirmed in these cells exhibiting increased expression ratio of Vimentin/CDH1. The HMLE cells treated with the ECM1^+^ exosomes also exhibited augmented production of Glutamate (p=0.001) compared to the control cells (Figure 3F). However, the HMLE cells treated with the sera exosomes obtained from the healthy or the NTM_nb_ subject did not exhibit the EMT phenotype or associated changes in the expression ratio of Vimentin/CDH1, when compared (Supplementary Figure 3).

### Influence of exosomal ECM1 on T cell activity

**Figure 4:**
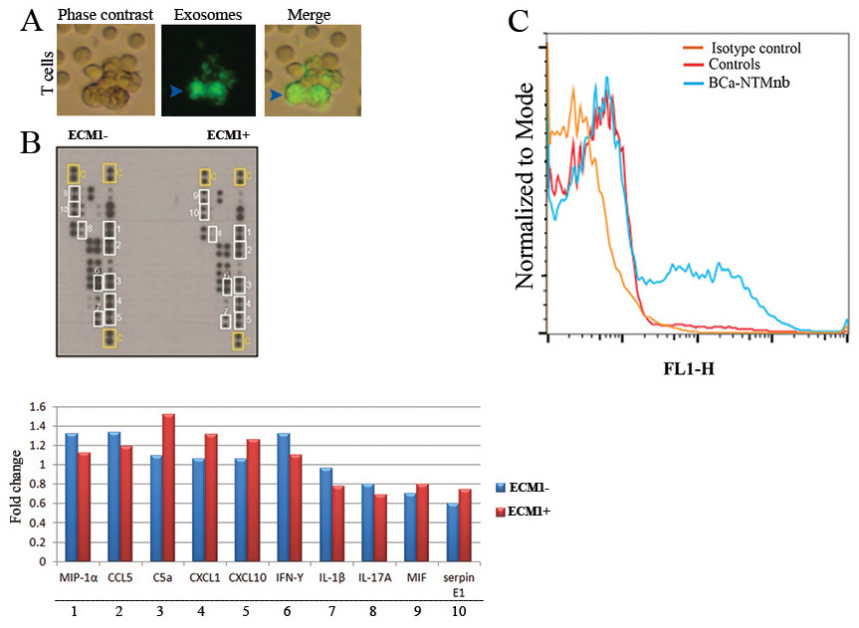
Cytokine profiling and exosome production analysis **A**. Uptake of the exosomes (arrowheads) by normal human T cells. **B**. Increased production of C5a, CXCL1, CXCL10, MIF and Serpin E1and decreased expression of MIF-1α, CCL5, IFN-γ, IL1β, and IL-17 in the human T cell culture with ECM1^+^ exosomes compared to the ECM1^-^ pool. The T cells were incubated for 1 hour with the various exosome pools followed by their activation for 48 hours as described under the method section. The expression data (fold change) for each cytokine was normalized to the normal controls in the array blot using the Image J software (NIH). The number corresponds to the respective cytokine in the immunoblot. **C**. The frequency (%) of ECM1 positive circulating exosomes (A, blue) was higher (p=0.0017) in the breast cancer patients with Bronchiectasis non tuberculous Mycobacterium (BCa-NTM_nb_) infection compared to the healthy or Bronchiectasis non tuberculous Mycobacterium (NTM_nb_)subjects (red). Rabbit IgG-FITC was used as the isotype control (orange).

To determine the impact of ECM1 on immune system function, we co-cultured normal human T cells and ECM1^+^ and ECM1^-^ exosomes obtained from the sera of one ECM1 positive BCa-NTM_nb_ case for 1 hour. We confirmed the exosomal uptake of the T cells (Figure 4A). We observed an increased expression of C5a, CXCL1, CXCL10, MIF and Serpin E1 (a.k.a. PAI-1) and decreased expression of MIF-1α, CCL5, IFN-γ, IL1β, and IL-17 in the human T cell culture incubated with ECM1^+^ exosomes compared to the ECM1^-^ exosome pool (Figure 4B). The normal human T cells co-cultured with the sera exosomes obtained from the healthy or the NTM_nb_ subject did not exhibit any considerable change in the cytokine expression, when compared (Supplementary Figure 4).

### Enrichment of ECM1^+^ exosomes in the BCa-NTM subjects

To determine the frequency of ECM1 positive exosomes in the circulation of the BCa-NTM subjects, we performed FACS analysis. The frequency of the ECM1^+^ exosomes was significantly higher (p=0.0017) in the BCa-NTM_nb_ subject (N=18) compared to the controls (N=14; 9 healthy and 5 NTM_nb_ subjects) (Figure 4C).

### Expression of APOC4, adiponectin, and AZGP1 in the circulating exosomes of the BCa-NTM subjects

**Figure 5:**
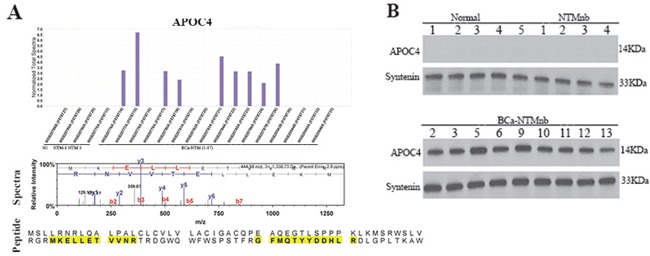
APOC4 detection and validation **A**. Mass spectroscopy analysis detected APOC4 expression only in the breast cancer patients with nontuberculous Mycobacterium (BCa-NTM) infection, but not in the healthy, normal (N1) or individuals with nontuberculous Mycobacterium (NTM) infection. The spectra (panel 2) and the 2 peptide sequences (shaded yellow, panel 3) of APOC4 detected in the exosomes. **B**. Western blotting of APOC4 in the circulating exosomes of the breast cancer patients with nontuberculous Mycobacterium (BCa-NTM) infection, healthy, normal (N) or individuals with nontuberculous Mycobacterium (NTM_nb_) infection. Syntenin was used as an exosome marker and loading control.

**Figure 6:**
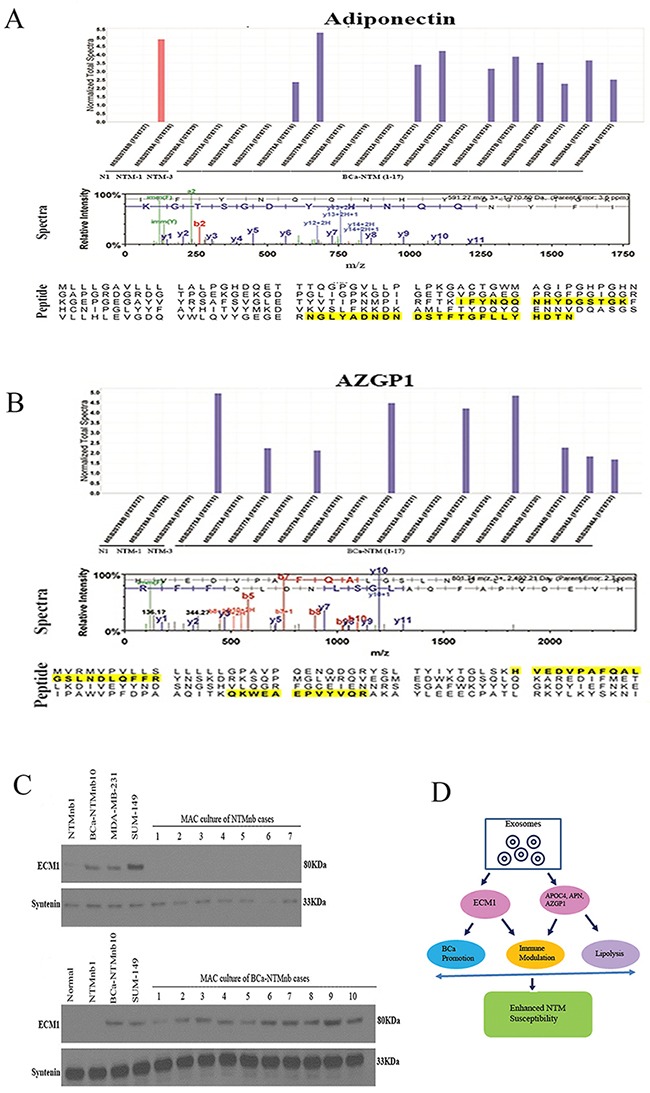
Secretome analysis in sera and MAC culture Mass spectroscopy analysis exclusively detected APN **A**. or AZGP1 **B**. expression only in the breast cancer patients with nontuberculous Mycobacterium (BCa-NTM_nb_) infection. The spectra (panel 2) and 2 unique peptide sequences of APN (a, shaded yellow, panel 3) and AZGP1 (b, yellow, panel 3) was detected in the exosomes. **C**. ECM1 expression was not detectable in the MAC culture derived exosomes of NTM_nb_ cases (N=7) but detectable in all BCa-NTM_nb_ subjects (N=10). Human breast cancer cell lines MDA-MB-231, SUM-149 and one BCa-NTM_nb_ subject were used as positive control for ECM1 detection. One healthy and one NTM_nb_ subject were used as negative controls. Syntenin was used as an exosome marker and loading control. **D**. Model suggesting the possible role of ECM1, APOC4, APN and AZGP1. Exosome derived ECM1 in concert with other secretory and lypolysis associated proteins APOC4, APN and AZGP1 might have influenced BCa development and immune suppression leading to increased NTM susceptibility.

We observed an exclusive expression of APOC4 protein in the circulating exosomes of 53% (9/17) of the BCa-NTM cases (Figure 5A). Similar to ECM1, APOC4 expression was not detected in the healthy control or NTM_nb_ subjects (Figure 5A). A number of unique spectra and a couple of unique peptides (Figure 5A) of APOC4 were detected in the circulating exosomes of the 9 BCa-NTM cases. Western blotting analysis using the exosome proteins from healthy normal, NTM_nb_ and the same BCa-NTM_nb_ subjects further confirmed the exclusive expression of APOC4 in the BCa-NTM subjects (Figure 5B). Additionally, exclusive expression of APN and AZGP1 was detected in the circulating exosomes of 59% (10/17) and 53% (9/17) of the BCa-NTM_nb_ cases respectively (Figure 6A–6B, Table 1). Adiponectin expression was also detected in 1 NTM subject (Figure 6, Table 1, NTM_nb_ 3). A number of unique spectra and a couple of unique peptides (Figures 6A-B) of APN and AZGP1 were detected in the circulating exosomes of the BCa-NTM_nb_ subjects.

### Correlation between ECM1, APN, AZGP1 expression and BMI of the BCa-NTM subjects

Among the 19 BCa-NTM_nb_ cases, 5 subjects were overweight or obese (BMI range 25-37.6) and 2 were underweight (BMI range 15.1-16.1) (Table 1). Eighty percent (4/5) of these higher BMI subjects had ECM1 but no APN or AZGP1 expression in their circulating exosomes (Table 1). On the other hand, the low BMI subjects had high ECM1 along with high APN expression in their circulating exosomes. One of these subjects (BCa-NTM 13) has been detected with high ECM1 and APN but no AZGP1 in the exosomes (Table 1). The single NTM_nb_ subject with high APN expression had a low BMI (18.4). There were 10 BCa-NTM_nb_subjects with normal BMI (18.5-23.8, Table 1). Expression of APN and AZGP1 was detected in 60% (6/10) and 70% (7/10) of these subjects respectively (Table 1). Co-expression of both APN and AZGP1 was detected in 40% (4/10) of these subjects.

### Mycobacterium avium complex culture derived exosomes from BCa-NTM_nb_ patients harbor human ECM1 protein

All the NTM and BCa-NTM subjects in our study were confirmed to have MAC infection. We determined the expression pattern of ECM1 in the exosomes derived from the MAC culture of women with NTM_nb_ (N=7) and BCa-NTM_nb_ (N=10). We could not detect ECM1 expression in the MAC culture derived exosomes of any of the NTM_nb_ cases (N=7, Figure 6C). However, ECM1 expression was detectable in the MAC culture derived exosomes of 100% (10/10) women with BCa-NTM_nb_ (N=10, Figure 6C).

### Analysis of the body morphotye

The BMI of the NTM_nb_ subjects was significantly lower (p=0.033) compared to the normal healthy women (Supplementary Figure 5). The BMI of the BCa-NTM_nb_ subjects were also significantly lower compared to the healthy control (p=0.019) but higher (p=0.001) compared to the NTM_nb_ subjects.

## DISCUSSION

Molecular factors predisposing and/or facilitating NTM infection in women with BCa are unknown. Preexisting and recurring genetic changes promoting immunosuppression and influencing the BMI could be associated with increased susceptibility to NTM disease. These abnormal genetic messages may frequently transport to neighboring cells through the circulating exosomes and influence tumorigenesis, immune function and microbial infection [5–6, 10–12]. Circulating exosome profiling could be an invaluable tool to identify the molecular footprints associated with the pathogenesis and progression of various diseases, including bronchiectasis and NTM lung disease. Through an MS based approach, we confirmed ECM1 as an exclusive and abundantly expressed protein in the circulating exosomes of BCa-NTM_nb_ and BCa subjects. Thus, ECM1 appears to be a BCa associated molecule abundantly present in the circulating exosomes. ECM1 is a membrane bound and secreted glycoprotein mapped to human chromosome 1q21 (Gene ID: 1893), a frequently amplified region in BCa [13].

Increased production of exosomes harboring growth promoting and immunomodulatory proteins, and their transport to the recipient cells could potentially favor both tumor growth and immunosuppression. Remarkably, irrespective of the mixed resources of the exosomes in circulation, we observed an ECM1 enriched exosome pool, which may have a role in NTM_nb_ development in these BCa subjects. In a recent study, ECM1 was shown to enhance the proliferation of breast cancer cells [14]. The delivery of ECM1enriched exosomes not only induced tumorigenic mammary epithelial cell development, but also modulated immune function. Various recent studies demonstrated the influence of exosomes on promoting tumor growth or modulating immune function [8, 12]. It remains to be determined how ECM1 was released by the exosomes after entering the cells. It is possible that by harboring numerous surface proteins, the exosomes could engage various cellular receptors as a mechanism of cargo delivery or uptake [5]. Studies have demonstrated the multidisciplinary function of ECM1 in BCa progression [14–18]. ECM1 was shown to disrupt T cell function through a direct interaction with IL-2 receptor and its depletion from the T cells impaired T_H_2 response and reduced allergic airway inflammation *in vivo* [19]. Thus, the release of high amounts of ECM1 in the circulation and their transport through the exosome cargo might have generated an immunosuppressive environment by impairing T cell and other immune cell function including alveolar macrophages, the primary site of pulmonary NTM infection [2]. Possibly as a reason, ECM1^+^ exosomes induced the production of various immunosuppressive cytokines [20–25] by the normal human T cells within an hour of treatment. Strikingly, exclusive ECM1 detection in the MAC derived exosomes from BCa-NTM_nb_ subjects suggests a role of ECM1 in facilitating immunosuppression or cellular transformation through the mycobacterium and their secreted exosomes. Collectively, these results suggest a new function of ECM1 through the circulating exosomes in promoting cellular transformation and modulating immune surveillance. Of note, 26% (5/19) of the BCa-NTM_nb_ cases first developed NTM_nb_, followed by BCa within a span of 4-10 years and have high levels of ECM1 in their circulating exosomes. It is possible that early abundance and exosomal transport of ECM1 facilitated immunosuppression and cellular transformation in these subjects leading to NTM and BCa development or*vice versa* in concert with other molecular alterations.

We also confirmed the exclusive expression of APOC4 in the circulating exosomes of the BCa-NTM_nb_ subjects. APOC4 is a highly conserved lipid binding protein belonging to the apolipoprotein C family and play a critical role in lipid metabolism [30, 26]. The contribution of APOC4 in BCa-NTM_nb_ disease is currently unknown. We observed exclusive expression of APN and AZGP1 in the circulating exosomes of the BCa-NTM_nb_ subjects. APN is a highly secreted pleotropic hormone affecting various pulmonary cell types including alveolar macrophages [27]. APN level increases in lean or slender individuals and decreases in subjects with higher BMI [27]. An elevated serum level of APN was found in the patients with pulmonary NTM [28]. APN is also known to induce the production of various immunosuppressive cytokines and thus its upregulation may increase the susceptibility to NTM infection [28]. In a recent study, increased plasma APN level was shown to be associated with increased mortality among patients developing acute respiratory distress syndrome (ARDS) from extra-pulmonary etiologies [27]. In another study, enhanced APN expression was shown to induce severe allergy response by modulating T_H_1 to T_H_2 pathway [29]. Similar to APN, we also observed exclusive expression of AZGP1, another secretory glycoprotein [30] in the circulating exosomes of the BCa-NTM cases. AZGP1 is a multifunctional protein involved in lipid mobilization, lipolysis, immunoregulation and cancer cachexia [30]. Similar to APOC4, the role of AZGP1 in BCa-NTM_nb_ disease remains elusive. Interestingly, all but one BCa-NTM_nb_ cases with high BMI did not have APN in their exosomes and on the contrary, the subjects with low BMI had APN expression. These results support the hypothesis that the slender individuals with low BMI and higher serum APN level have an increased risk of NTM development [28, 31]. A similar correlation was evident with AZGP1 in these subjects as described above. On the other hand, the majority of the BCa-NTM_nb_ subjects with normal BMI also had APN and AZGP1 expression in their circulating exosomes. These results indicate the potential influence of these molecules towards NTM susceptibility irrespective of the BMI status. This notion is further supported by the fact that the BCa-NTM_nb_ cases with significantly higher BMI compared to the NTM_nb_ subjects expressed APN, AZGP1 in addition to ECM1. Collectively, these findings implicate the potential benefit of monitoring circulating exosomes in the routine clinical application. To our knowledge, no studies have so far reported the concurrent expression of ECM1, APN, AZGP1 and APOC4 and in the circulating exosomes of the BCa-NTM_nb_ subject.

Increased Glutamate production is associated with BCa progression and inhibition of Glutamate release appears to be a potential therapeutic target in BCa [32]. On the other hand, Glutamate is also produced by various types of immune cells, including neutrophils, monocyte/macrophages and T cells [33]. Moreover, Glutamate released by various immune cells binds to the Glutamate receptors (GluRs) and may augment or suppress the immune system in a context dependent manner [33]. Thus, higher amount of Glutamate production following ECM1 treatment might be associated with immune modulation as well. However, it remains to be determined, how the exosome-ECM1signaling induced Glutamate production and leads to immunosuppression in the BCa-NTM_nb_ patients.

In summary, our study suggests that the BCa associated secretory protein ECM1, being abundantly present and transported through the circulating exosomes, modulates immune function leading to increased susceptibility to NTM disease (Figure 6D). On the other hand, APOC4, APN and AZPG1 as additive factors might possibly enhance NTM susceptibility thorough the modulation of immune function and triggering lipolysis (Figure 6D). In the clinical settings, a large scale and comprehensive analysis of ECM1, APOC4, APN and AZGP1 in the circulating exosomes is warranted, which may be beneficial for risk assessment and monitoring of BCa patients towards developing NTM disease.

## MATERIALS AND METHODS

### Human samples and ethical statement

Serum samples were collected from normal, healthy women (N=5), women with NTM_nb_ (N=5) and women with BCa-NTM_nb_ (N=19) at The University of Texas Health Science Center at Tyler under an IRB approved protocol (#974). All patients were de-identified and only relevant clinical information such as age, grade, stage, diagnosis etc. was collected. All cases except for the normal subjects were tested positive for *Mycobacterium avium* complex infection by acid fast sputum analysis. The relevant demographic information of all subjects was presented in Tables 1, 2. We have also collected archived sera samples from 40 de-identified BCa subject from the Cooperative Human Tissue Network (CHTN) through an IRB approved protocol ((#959).

**Table 2:** Demographic information of the NTM_nb_ subjects with corresponding exosome protein expression

^1^Patient ID	Age at NTM Diagnosis	^2^Smoking/Drinking	Weight (lb)	Height (inches)	^3^BMI	^4^ECM1	APN	AZGP1	APOC4
1	49	N/Y	109	65	*18.1*	-	-	-	-
2	63	Y/Y	122	-		-	-	-	-
3	58	Y/Y	114	66	*18.4*	-	+	**-**	**-**
4	69	N/N	108	63	19.1	-	-	-	-
5	62	N/N	130	65	21.6	-	-	-	-

^1^Women with Bronchietasis Nontuberculous Mycobacterium infection only. ^2^History of tobacco smoking and alcohol drinking. Y: Yes; N: No. ^3^BMI: Body mass index was determined following NHLBI criteria: Underweight = <18.5; Normal weight = 18.5–24.9; Overweight = 25–29.9; Obesity = BMI of 30 or greater. ^4^Expression status of the ECM1, APN, AZGP1 and APOC4 in the circulating exosomes.

### Body mass index

The Body Mass Index (BMI) was determined following the criteria of the National Heart, Lung and blood Institute: Underweight = <18.5; Normal weight = 18.5–24.9; Overweight = 25–29.9 and Obesity = BMI of 30 or greater.

(http://www.nhlbi.nih.gov/health/educational/lose_wt/BMI/bmicalc.htm)

### Antibodies and reagents

The ECM1 (# NBP229464) and APOC4 (#H00000346-B01) antibodies were purchased from Novus Biologicals. The ECM1-FITC and rabbit-IgG-FITC antibodies (#orb15539; # orb248103) were procured from Biorbyt Inc. The Adiponectin (#2789S), CDH1 (#3195) and Vimentin (#5741) antibodies were from Cell Signaling Inc. and the AZGP1 antibody was procured from Abcam Inc. (#ab117275). The Syntenin antibody (#H00006386-M01) was procured from Abnova Inc. Anti-mouse (#115-035-003) and rabbit (#111-035-003) secondary antibodies were obtained from Jackson Immunoresearch. The HMLE and SUM149 cell lines were received from Dr. Giojun Wu, Wayne State University and MDA-MB-231 from ATCC.

### Exosome preparation

Exosomes were isolated from human sera followed by protein isolation as described [11]. The human breast epithelial cell lines were cultured for 1 week in medium containing exosome depleted FBS (#EXO-FBS/HIxxx, System Biosciences). After 1 week, the exosomes were isolated from these cell lines as described [11–12]. We also isolated exosomes from the sputum derived MAC cultures using the above described kit (# EXOTCxxA-1) and protocol [11].

### Nanosight tracking of the exosomes

Exosome suspension was gently vortexed at 2.5k for 10 seconds and then bath sonicated for 10 min at 33°C. NanoSight measurements were carried out in 0.02 um filtered PBS and then visualized on an LM10 NanoSight instrument (Supplementary Figure 6). The exosome samples were visualized and assessed for the purity and size (Supplementary Figure 6).

### Sample preparation for mass spectroscopy

To the exosome pellet, 50μL of 10% SDS was added (2% v/v) and then heated at 95°C for 15 minutes followed by protein isolation. Protein concentration was determined using a Qubit fluorimeter assay (Invitrogen). The protein samples were then run in 10% Bis-Tris homogeneous SDS-PAGE gel followed by band excision, trypsin (Promega) digestion at 37°C for 4h and quenching with formic acid. The supernatant was analyzed directly without further processing.

### Mass spectrometry

Each digested sample was analyzed by nano LC/MS/MS with a Proxeon EASY-nLC 1000 HPLC system interfaced to a ThermoFisher Q Exactive mass spectrometer. Thirty microliter of sample was loaded on a trapping column and eluted over a 50μm x 150mm analytical column (Thermo Fisher P/N ES-801) at 300nL/min using a 2hr reverse phase gradient; both columns were packed with PepMap RSLC C18, 2 μm resin (Thermo Scientific). The fifteen most abundant ions were selected for MS/MS.

### Data processing and database searching

Data were searched using a local copy of Mascot ((Matrix Science, London, UK; version 2.5.1) with the following parameters: Enzyme: Trypsin/P; Database: Swissprot Human (concatenated forward and reverse plus common contaminants. Fixed modifications: Carbamidomethyl (C); Variable modifications: Oxidation (M), Acetyl (N-term), Pyro-Glu (N-term Q), Deamidation (N,Q); Mass values: Monoisotopic Peptide Mass Tolerance: 10 ppm; Fragment Mass Tolerance: 0.02 Da Max Missed Cleavages: 2. Mascot DAT files were parsed into the Scaffold software for validation, filtering and to create a non-redundant list per sample. Data were filtered using at 1% protein and peptide FDR and requiring at least two unique peptides per protein. The Mascot was set up to search the SP-Human-012015_012015 database (unknown version, 40656 entries) assuming the digestion enzyme strict trypsin.

### Criteria for protein identification

Scaffold (version Scaffold_4.4.5, Proteome Software Inc., Portland, OR) was used to validate

MS/MS based peptide and protein identifications. Peptide identifications were accepted if they could be established at greater than 92.0% probability to achieve an FDR less than 1.0% by the Scaffold Local FDR algorithm. Protein identifications were accepted if they could be established at greater than 99.0% probability to achieve an FDR less than 1.0% and contained at least 2 identified peptides. Protein probabilities were assigned by the Protein Prophet algorithm [34].

### Western blotting and immunofluorescence analyses

Western blotting was performed as described [11]. In addition to Syntenin, CD63 was used as another exosome marker (Supplementary Figure 1B) [35]. The immunofluorescence (IF) analysis was performed as described [35].

### Co-culture of normal human breast epithelial cells and the exosomes

High ECM1 expressing exosomes were enriched from the serum of one BCa-NTM_nb_ subject as follows. Exosomes were incubated with a FITC labelled anti-human ECM1 antibody for 30 minutes at 4°C. The FITC-ECM1 positive exosomes were then purified using anti-FITC magnetic beads (#130-048-701, Miltenyi Biotech) followed by FACS analysis. The ECM1^-^ exosomes from the same subject were used as a control. The HMLE cells were then co-cultured with ECM1^+^ and ECM1^-^ exosome pool for 7 days [11]. The HMLE cells were cultured in media depleted of exosomes [11]. A similar experiment was performed with the exosomes isolated from one healthy and one NTMnb subject as controls.

### Epithelial to mesenchymal transition and glutamate analyses

Epithelial to mesenchymal transition (EMT) of the exosome treated cells was determined as described [35]. Glutamate production of the exosome treated cells was measured using the YSI 2900D Biochemistry Analyzer (Yellow Spring Instruments) as described [36].

### Human T cell co-culture with the exosomes and cytokine profiling

We isolated human T cells by the human pan T cell Isolation kit II (Miltenyi Biotec #130-091-156). For T cell activation, we first coated a 96-well plate with 50 ul/ well sterile PBS containing anti-CD3 mAb at 5 ug/ml and anti-CD28 at 1 ug/ml and incubated for 2 hours at 37°C. During this time, we treated the T cells with ECM1^+^ and ECM1^-^ exosomes (1×10^6^) for 1 hour before stimulation with plate bound anti-CD3 and anti-CD28 antibodies. The T cells were then added at a concentration of 2 × 10^5^ in 200 ul /well in complete RPMI-1640 with 10% human AB serum in duplicates. The cells were incubated for 48 hours, supernatants were collected and analyzed for the expression of 40 cytokines using a cytokine detection kit and protocol (# ARY005B, R&D Systems). A similar experiment was performed with the exosomes isolated from one healthy and one NTMnb subject as controls.

### FACS analysis

To determine ECM1 positive exosome population, we isolated the exosomes from sera.. The exosomes were incubated with an anti-human ECM1-FITC antibody for 30 minutes at 4°C. The exosomes were then washed and finally suspended in 500 μl of FACS buffer and analyzed in a BD FACScanner [37]. We used a FITC conjugated rabbit-IgG as an isotype control.

### Statistical analysis

Chi-square, Fisher's exact or Student's *t* tests were used when appropriate. All p-values were two-sided and all confidence intervals were at the 95% level. Computation for all the analysis was performed using the Statistical Analysis System (SAS).

## SUPPLEMENTARY MATERIALS FIGURES AND TABLES








