# Supplementary material for: Exosome secretome and mediated signaling in breast cancer patients with nontuberculous mycobacterial disease
Source: Oncotarget. 2017 Feb 1;8(11):18070–81. doi: 10.18632/oncotarget.14964 (PMC5392308; doi:10.18632/oncotarget.14964)
Supplement: Supplementary file 1 [file oncotarget-08-18070-s001.pdf]

Exosome secretome and mediated signaling in breast cancer patients with nontuberculous mycobacterial disease

SUPPLEMENTARY FIGURES AND TABLES

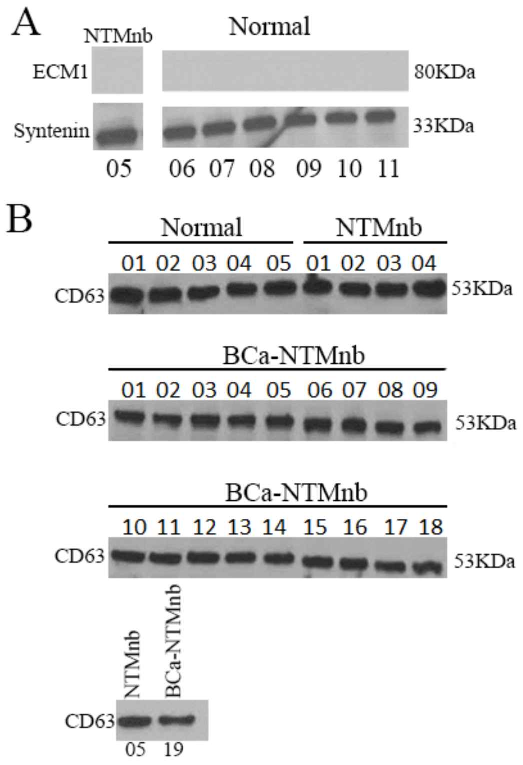

Supplementary Figure 1: A. Western blot analysis of ECM1 in additional normal and NTM_{nb} subjects. Syntenin was used as an exosome marker. **B.** Western blot analysis of the sera exosomes with the exosome marker CD63 from various designated groups as indicated.

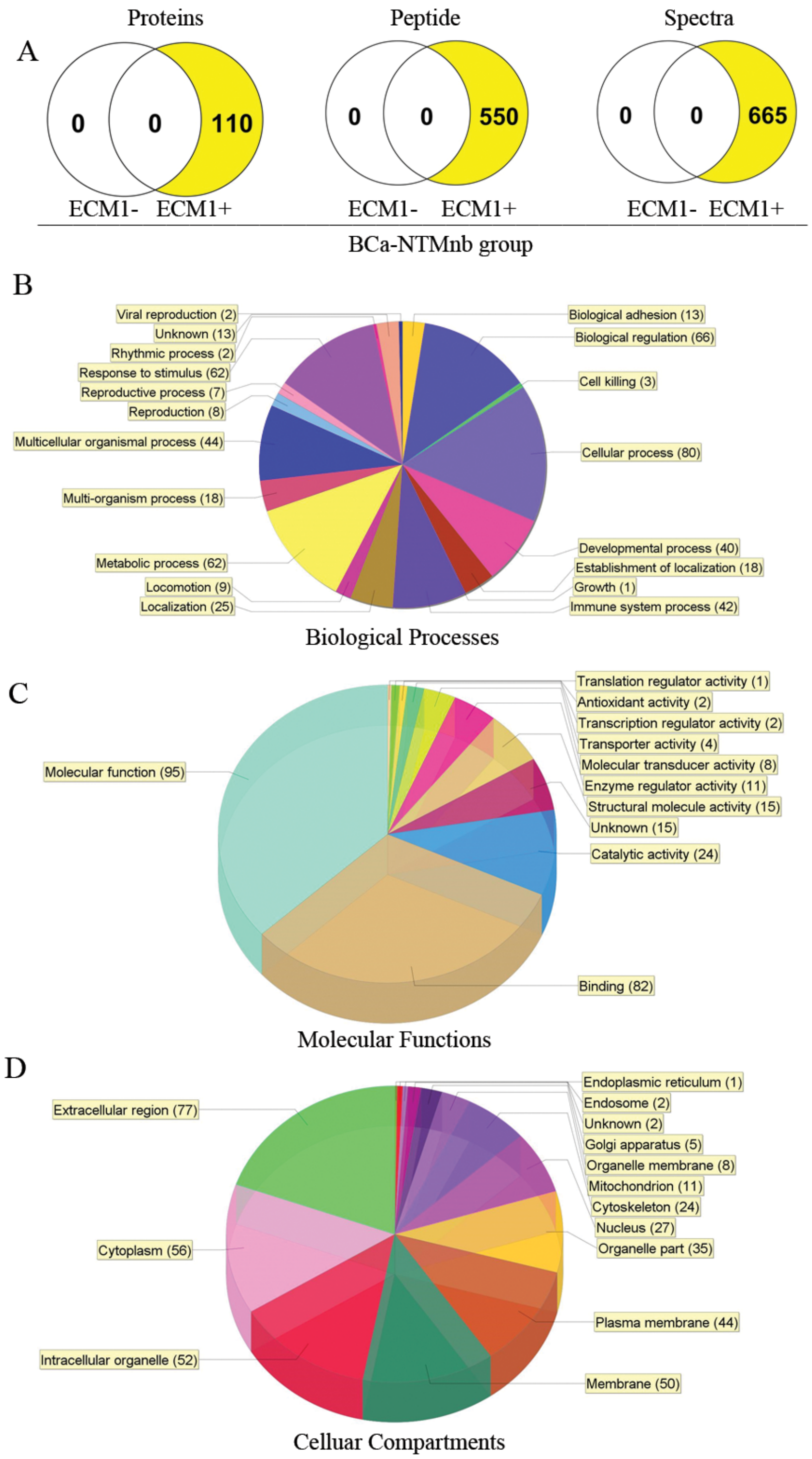

Supplementary Figure 2: Exosome proteome profiling in ECM1^{+} vs. ECM1^{-} BCa-NTM$_{nb}$ subjects. A. Venn diagram showing exclusive signature of 110 proteins, their unique peptides and spectra detected in the circulating exosomes of the ECM1^{+} BCa-NTM$_{nb}$ subjects (yellow shades) compared to the ECM1^{-} ones. **B-D.** Pie charts exhibiting localizations and molecular functions of these unique proteins regulating various biological pathways.

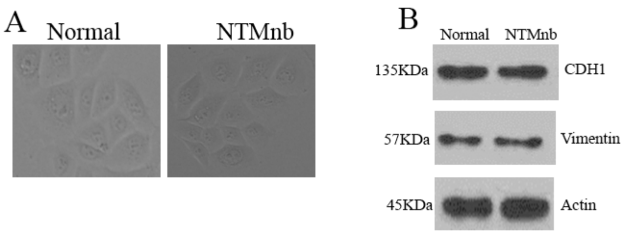

Supplementary Figure 3: Epithelial to mesenchymal transition analysis. HMLE cells were co-cultured with the exosomes isolated from one healthy as well as one NTM_{nb} subject. No considerable changes in **A.** EMT or **B.** Vimentin/CDH1 expression ratio between healthy and NTM_{nb} groups. Magnification X 200 (A). **C.** Actin was used as loading control in the Western blot analysis (B).

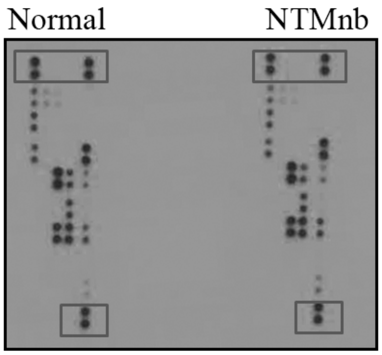

Supplementary Figure 4: Cytokine production of normal human T cells treated with control exosomes. Normal human T cells were incubated for 1 hour with the exosomes prepared from one healthy and one NTM_{nb} subject followed by their activation for 48 hours as described under the method section. No considerable changes in cytokine production between groups treated with exosomes from the healthy or NTM_{nb} subject. Rectangular blocks indicate control cytokine spots.

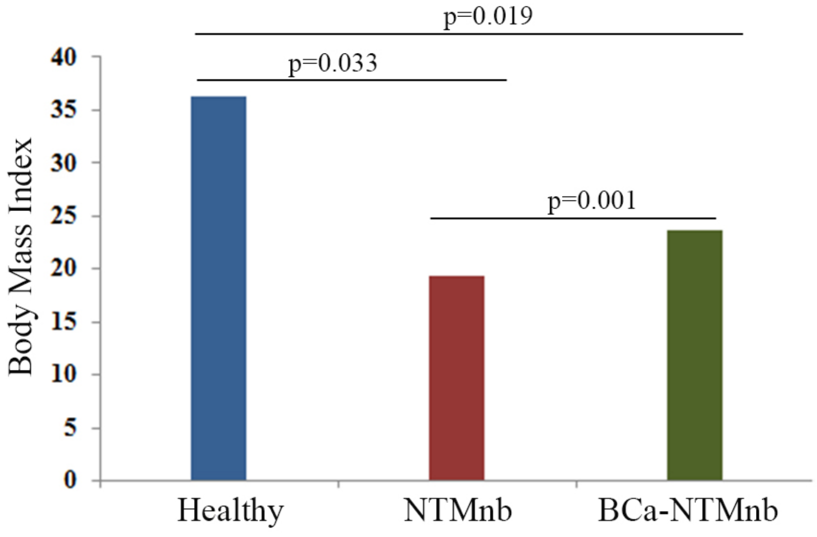

Supplementary Figure 5: The BMI of the NTM_{nb} subjects was significantly lower (p=0. 033) compared to the healthy subjects. The BMI of the BCa-NTM_{nb} cases was also significantly lower (p=0.019) compared to the healthy subjects. The BMI of the BCa-NTM_{nb} cases was significantly higher (p=0.001) compared to the NTM_{nb} subjects.

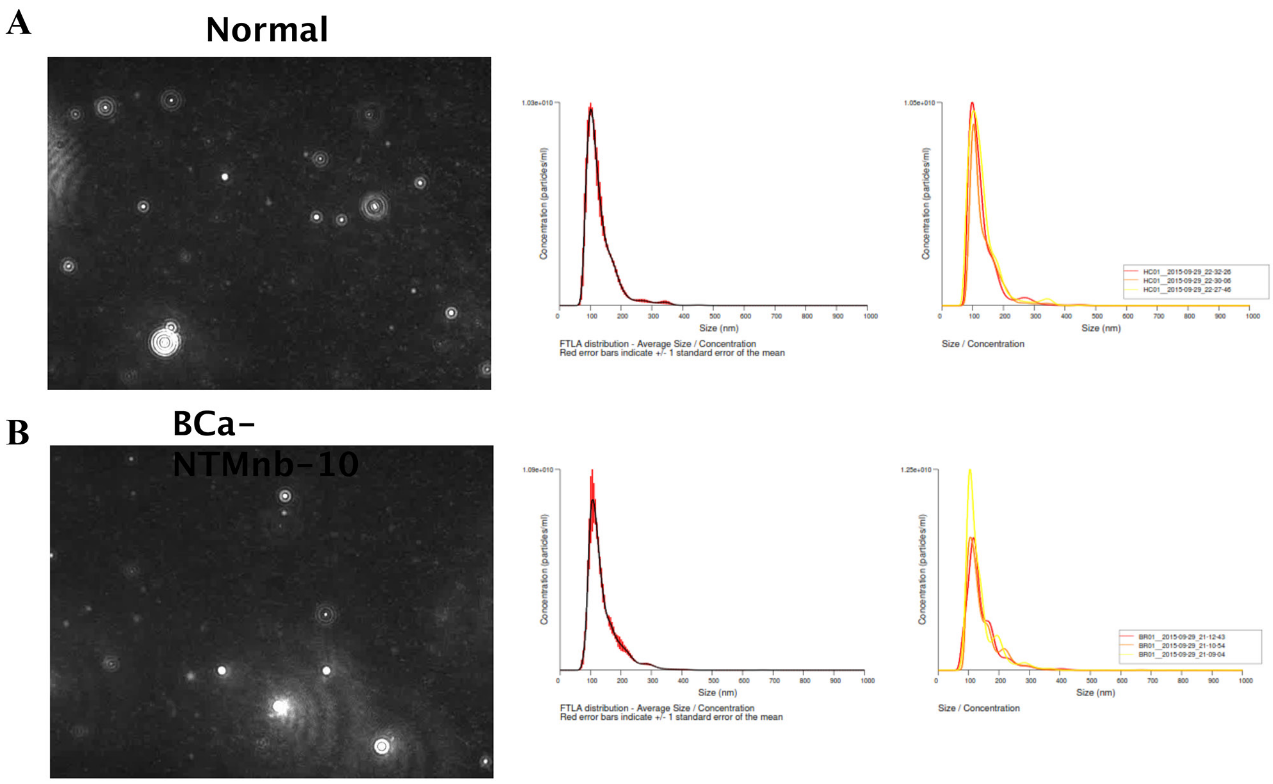

Supplementary Figure 6: (video). Nanosight tracking of the circulating exosomes from a healthy normal control **A.** and a breast cancer patient **B.** with nonmycobacterium tuberculous (NTM) infection. The diagram (right panel) shows the size range of the exosomes.

Supplementary Table 1: List of all the 350 proteins, the number of their unique spectra and peptides detected in the circulating exosomes of various study populations. Healthy subject: 20788; NTM_{nb} subjects: 20789 (NTM_{nb}1) and 20790 (NTM_{nb}3). BCa-NTM_{nb} subjects: 20775-20787 and 20943-20946.

See Supplementary File 1

Supplementary Table 2: List of the 118 signature proteins detected exclusively in the circulating exosomes of the BCa-NTM_{nb} subjects.

See Supplementary File 2

Supplementary Table 3: List of the 110 proteins detected exclusively in the circulating exosomes of the $ECM1^+$ BCa-NTM_{nb} subjects (N=14) compared to the $ECM1^-$ 3 (N=3) BCa-NTM_{nb} subjects.

See Supplementary File 3
